# Readiness for advance care planning and related factors in the general population: a cross sectional study in Iran

**DOI:** 10.1186/s12904-024-01496-2

**Published:** 2024-07-09

**Authors:** Ali Askari, Hosein Mohammadi Roshan, Nasim Abbaszadeh, Mahmood Salesi, Seyed Morteza Hosseini, Mobina Golmohammadi, Salman Barasteh, Omid Nademi, Razieh Mashayekh, Mohammad Hossein Sadeghi

**Affiliations:** 1 Medicine, Quran and Hadith Research Center, Baqiyatallah University of Medical Sciences, Tehran, Iran; 2 Nursing Faculty, Baqiyatallah University of Medical Sciences, Tehran, Iran; 3 Student Research Committee, Baqiyatallah University of Medical Sciences, Tehran, Iran; 4 Chemical Injuries Research Center, Systems Biology and Poisonings Institute, Baqiyatallah University of Medical Sciences, Tehran, Iran; 5 Nursing Care Research Center, Clinical Sciences Institute, Baqiyatallah University of Medical Sciences, Tehran, Iran

**Keywords:** Advance care planning, Advance directives, Iran, Palliative care, Hospice, End of life care, Living will

## Abstract

**Context:**

Advance Care Planning (ACP), as a process for expressing and recording patients' preferences about end-of-life care, has received increasing attention in recent years. However, implementing ACP has been challenging in Iran.

**Objectives:**

To assess the readiness for advance care planning and related factors in the general population of Iran.

**Methods:**

This cross-sectional study was conducted on the general population of Iran in 2022. The data was collected using demographic information questionnaire and The RACP Scale. The purpose and methodology of the research was explained to all participants, and upon their agreement an informed consent was obtained. Participants were invited to fill out the questionnaires wherever is more convenient for them, either alone or if needed, with the help of the researcher to protect their privacy. Chi-square, fisher exact test and multiple logistic Regression model were used to assess the effective factors on the RACP. The data were analyzed by SPSS software version 26.

**Results:**

A total of 641 people with an average age of 36.85 ± 12.05 years participated in this study. Of those, 377 (58.8%) had high RACP. The logistics model showed an association between the chance of readiness for receiving ACP with participants’ education level, such that the chance of readiness in those with Master's or Ph.D. degrees was three times higher than those with a diploma (*p* = 0.00, OR:3.178(1.672, 6.043)). However, the chances of readiness in those with bachelor’s degrees was not significantly different from those with a diploma (*p* = 0.936, OR: 0.984 (0.654, 1.479)). Moreover, the chance of readiness was 1.5 higher in participants over 40 years of age compared with participants under the age of 40 (*P* = 0.01, OR: 1.571(1.10, 2.23)).

**Conclusion:**

According to the findings of this study, it can be concluded that there is a relatively RACP among people in Iranian society. The readiness of individuals for ACP increases by their age and education level. Therefore, by holding appropriate training intervention, we can increase the readiness of the public for ACP to improve their end-of-life outcome.

**Supplementary Information:**

The online version contains supplementary material available at 10.1186/s12904-024-01496-2.

## Introduction

Each year, 41 million people die worldwide due to the rise in chronic diseases among ageing population. This number of deaths accounts for 71% of all deaths globally [1]. Several studies have shown that about 80% of deaths in low- and middle-income countries such as Iran are caused by non-communicable and chronic diseases [2]. Mortality from non-communicable diseases has increased by 14.5% in the past 20 years and the probability of premature death of an Iranian from one of the non-communicable diseases has been 17.3% [3]. In 2016, the mortality rate from non-communicable diseases in Iran is 304,400 [4]. According to the World Population Aging report in 2019, it is estimated that the population aged 65 and above will reach from 5,272(6.4%) in 2019 to 20,788(20.2%) per 100,000 in 2050 in Iran [5].

Given the two challenges mentioned (aging and the burden of chronic diseases), it appears that many people around the world are experiencing life-threatening conditions in their last years of their lives and require end-of-life care [6]. As a moral principle, these people have the right to receive end-of-life care tailored to their preferences, interests, values and culture [7].

Communication about the patient’s preferences, interests, values and culture between patient, next of kin and health care personnel is known as advance care planning (ACP) and include setting priorities for end-of-life care of patients [8]. ACP has attracted a lot of attention around the world. ACP is a process by which people plan their future care by expressing their preferences and documenting them [9]. In a recent systematic review found that, ACP interventions improve quality of patient–physician communication, preference for comfort care, decisional conflict, patient- caregiver congruence in preference, and ACP documentation [10]. Also, ACP, aims to support person-centered medical decision-making based on patient preferences [11]. In ACP process patients can discussed regarding the use of life-sustaining technologies such as dialysis machines or ventilators, cardiopulmonary resuscitation (CPR), Artificial Nutrition (AN) and Palliative Care (PC) [12].

Studies in the area of ACP initiated in the United States [13]. For the first time, ACP based on Patient Autonomy was enacted in the United States in 1976 under the California Natural Death Act. This law outlined the right for those who wished to choose an alternative decision-maker or to document their medical preferences before losing their ability for decision–making [14]. Later, in 1990, the U.S. Congress enacted the Patient Self-Determination Act to encourage the use of advance directives (ADs) [15]. The law mandated recognition of patients' rights to refuse or accept treatment. Today, ACP is implemented in many countries such as the United States, Canada, Netherlands, Australia and China [16].

In recent years, there has been a high demand for research and clinical care in the area of ACP in Asia. However, the culture of decision making and medical legal systems in Western countries differs significantly from those in Asian countries [13]. In Asian and Islamic culture, most people are reluctant to talk about ACP and prefer to avoid having open-ended conversations about end-of-life care [17]. Another important cultural factor that affects ACP in Eastern societies is the contribution of family members in decision making about treatment and medical care, but in Western culture, the focus is on patient autonomy [18]. Moreover, some Islamic sources acknowledge the patients’ right about their end-of-life care, as such those who take care of a patient are not allowed to force him/her to undergo treatment. However, many Muslim doctors are not aware of this right and provide treatment until the last moments of their patients’ lives [19–21].

ACP is an exotic concept for Muslims that is rarely discussed [22]. However, several studies have examined the views of the Islamic community regarding ACP and revealed that people in the Muslim community are eager to learn about and are receptive to the concept of ACP [23]. In a study conducted in 2021 in Lebanon, 18.6% of participants were familiar with ACP and 77.2% were interested in documenting their health values and preferences. Women were also more aware of ACP than men. Men had higher willingness to perform life-prolonging interventions [24]. In the study by Yunus et al. 56.2% to 72.6% of American Muslims were in the pre-thinking stage about ACP and mainly were unprepared to interact with the concept of ACP. Participation in ACP was correlated with participants' age, ethnicity, duration of residence in the United States, and their country of birth [25].

Other studies in non-Islamic cultures have reported other effective factors concerning the readiness for ACP (RACP). In the study conducted by Black et al*.* in the USA, age and education level were found to be useful factors for improving the RACP [26]. In agreement, a study by Laura I. van Dyck et al. in the USA and Yappu et al. in Australia also indicated that education level is highly effective in RACP [27, 28]. A study in 2017 showed that 8.7% of Japanese had ADs conversations [29]. In the study performed by Park et al., 16% of the general population were aware of the previous guidelines and more than 63% of the general population were eager to implement ACP when their disease had escalated or ended [30]. In a scoping review study by Grant et al. in 2021, 9,800 participated in the research, of which 80–90% reported to have ACP awareness [31].

The readiness of patients for participation in ACP and speaking about death is an important issue, which if neglected, would seriously harm the patient [32]. The humans’ view about death and readiness or confrontation with our issues affected by various factors including religion, culture, and values [17]. For example, since the Westerners have been trained about death in their culture, they are far more willing to talk about death as compared to their Asian counterparts. In the Asian culture, most people get distressed when talking about death and prefer to refrain from open conversations about the issues related to the end of life [33]. Meanwhile, ACP attempts to prepare patients for death. Therefore, if patients enter discussion without acquiring the readiness, not only do they get harmed but also their needs would not be properly recognized by the healthcare providers, and they would not achieve ACP goals [34]. Simon et al. regarded the time of initiating ACP as a challenging issue and defined poor perception of disease by patients as one of the obstacles of ACP [35]. On the other hand, Brinkman-Soppelenburgh et al. in a systematic review stated that if involvement in the ACP process is stressful for patients, its implementation would not have any impact on compatibility of cares with the needs and satisfaction of patients as well as their families [36]. Another review indicated that the patients’ tendency to participate in ACP is different. Some are fearful to face death and think that if they talk about death with their family, they would impose great stress and burden on them. Thus, initiation of ACP within this time period aggravates the patient fear and distress, and would make the outcome of the program unpleasant for them [37].

In Iran, so far, studies have been conducted on people's end-of-life preferences, such as the preferred place of death [38], the attitude of doctors and nurses regarding the do-not-resuscitate order [39–41], as well as the psychometrics assessment of the advance care planning questionnaire to Persian [42]. Despite the efforts that have been made regarding advanced care planning in Iran, so far no study has been done to examine the readiness of the Iranian society regarding advanced care planning. Therefore, the present study aimed to assess the readiness for advance care planning and related factors in the general population in Iran.

## Methods

### Design and setting

This cross-sectional study assessed the factors associated with ACP in caregivers of the patients who visited Baqiyatallah outpatient clinic in Iran in 2022. Sampling setting was Baqiyatallah outpatient clinic. In this clinic, patients come with chronic diseases such as cardiovascular problems, blood pressure, diabetes, neurological, respiratory and rheumatology diseases.

### Participants and sampling method

The participants included the caregivers of the patients who visited Baqiyatallah outpatient clinic with their patients. 641 caregivers included by convenience sampling. Due to the lack of similar studies in Iran, taking into readiness level of 50%, the type I error of 5% and the absolute error of 4%, the sample size was estimated to be at least 600 samples. The response rate was 85% (Additional file 1). Participation in the study was voluntary and participants had the right to withdraw from the study at any time. They were assured that all their information remains confidential. They were invited to fill out questionnaires at any location they wanted, either alone or if needed, with the assistance of the researcher to protect their privacy. Inclusion criteria were as follows: Referred to Baqiyatallah Hospital as a companion of the patient, age above 18 years, reading and writing literacy in Persian, and written consent for participation in the study. Exclusion criteria included partial completion of the questionnaire and simultaneous participation in another study associated with ACP. None of the participants reported feeling uncomfortable while filling out the questionnaires.

## Instrument

### Demographic data

Demographic information included age, sex, marital status, number of children, education, salary satisfaction, religion, race, living status, employment status, experience of loss and family caregiving.

### Readiness for advance care planning scale

This instrument was developed in 2019 by Sakai et al. to assess the RACP on the general population in Japan. This instrument had 28 items that during the psychometric evaluation of Persian version, one item was omitted. The Persian version consists of 7 items about” Recognize the importance of talking and writing”, 4 items about” Intend to talk”, 4 items about “intend to write”, 4 items about” Preparations for the behavior” and 8 items about “Practice of talking and writing”. The answer to all items is based on Likert 6–point rating scale. The lowest score for each item is 1 (totally disagree) and the highest score is 6 (totally agree). Therefore, the lowest score of the questionnaire is 27 and the highest score is 162 [8]. In this study, initially a forward -backward translation permission was obtained from the instrument designers Sakai*.* Then face validity was conducted by 10 people. Content validity was also performed by 10 experts. During the exploratory factor analysis, the item 8 was removed due to low loading factor. Confirmatory factor analysis was confirmed the 5-factor model. Reliability of the instrument was performed using internal consistency method (α = 0.941). The reliability of test–retest stability was also defined (ICC = 0.714).

### Data collection

After explaining the purpose and methodology of the research, informed consent was obtained from all participants.

### Data analysis

After data collection, the collected data were analyzed by SPSS software version 26. Descriptive analyses including frequency and percentage were used for qualitative data, and mean and standard deviation were used for normal quantitative data. We consider RACP as outcome variable. We considered the number 104 (median scores answered to RACP) as the cutoff point. In this way, we considered scores higher than 104 as high RACP and scores lower than 104 as low RACP. The correlation between the RACP with demographic factors of the subjects were examined using chi-square and Fisher exact tests. The threshold for statistical significance was 0.05. Finally, significant variables were included in the model through multiple logistic regression with the Wald backward method. The effect of individual explanatory variables on the outcome variable was measured using the adjusted odds ratio (AOR) with a 95% confidence interval (CI).

## Result

### Demographic characteristics

In this study, 641 members of the community participated. The mean age of the participants was 36.85 ± 12.05, of which 427 (66.6%) were under 40 years old. 382 (59.6%) of the participants were female and the rest were male. A total of 131 (20.4%) had diplomas, 425 (66.3%) bachelors and 85 (13.3%) had master's or doctoral degrees. Approximately 91 (14.2%) reported to live alone and 550 (85.8%) with their family. 482 (74.9%) participants were employed and 161 (25.1%) were unemployed (Table 1).

**Table 1:** Demographic information

Variable	Categories	*n*	%
**Gender**	Male	259	40.4
	Female	382	59.6
**Age**	40 >	427	66.6
	> 40	214	33.4
**Marital**	Single	206	32.1
	Married	401	62.6
	widowed/ Divorced	34	5.3
**Education**	Diploma	131	20.4
	Bachelor's	425	66.3
	Master's Degree/ Ph.D	85	13.3
**Income**	High Satisfaction	60	9.4
	Average Satisfaction	329	51.3
	Dissatisfied	252	39.3
**Live**	Single	91	14.2
	With Family	550	85.8
**Job**	Employed	480	74.9
	Unemployed	161	25.1
**Death Experience**	Yes	202	31.5
	No	439	68.5
**Caring Experience**	Yes	327	51
	No	314	49

### Factors related to advance care planning

Out of 641 participants, 377 (58.8%) had high readiness and 264 (41.2%) had low RACP. Statistical tests from chi-squared test showed no significant differences between RACP with gender, marital status, income, living condition, employment status or experience of loss or family caregiving. However, there was a significant difference between the level of education with RACP, such that people with Master's or Ph.D. degree had a higher RACP (*p* = 0.00) compared to those with a diploma or bachelor's degree. Besides, there was a substantial statistical correlation between age and RACP. As such the RACP was greater in participants over the age of 40 (*P* = 0.01) (Table 2).

**Table 2:** Correlations between variables and RACP

Variable	Categories	low	high	*P*-value
**Gender**	Male	113(43.6%)	146(56.4%)	0.30
	Female	151(39.5%)	231(60.5%)	
**Marital**	Single	95(46.1%)	111(53.9%)	0.13
	Married	153(38.2%)	248(61.8%)	
	Death of Wife/ Divorced	16(47.1%)	18(52.9%)	
**Income**	High Satisfaction	19(31.7%)	41(68.3%)	0.21
	Average Satisfaction	134(40.7%)	195(59.3%)	
	Dissatisfied	111(44%)	141(56%)	
**Live**	Single	44(48.4%)	47(51.6%)	0.13
	With Family	220(40%)	330(60%)	
**Death Experience**	Yes	84(41.6%)	118(58.4%)	0.88
	No	180(41%)	259(59%)	
**Caring Experience**	Yes	135(41.3%)	192(58.7%)	0.95
	No	129(41.1%)	185(58.9%)	
**Education**	Diploma	55(42%)	76(58%)	0.00
	Bachelor's	192(45.2%)	233(54.8%)	
	Master's Degree/ Ph.D	17(20%)	68(80%)	
**Age**	40 >	191(44.7%)	236(55.3%)	0.01
	> 40	73(34.1%)	141(65.9%)	
**job situation**	Employed	195(40.6%)	285(59.4%)	0.61
	Unemployed	69(42.9%)	92(57.1%)	

### The logistics model of factor related to advance care planning

Generated based on the Backward Walt method.. Individuals with a Master's / Ph.D. degree were three times more likely to be prepared than those with a diploma (*p* = 0.00, OR: 3.178(1.67, 6.04)). The chances of readiness for those with a diploma were not significantly different from those with a bachelor's degree (*p* = 0.93, OR: 0.98 (0.65, 1.47)). The chance of high RACP in participants over the age of 40 was 1.5 fold more than those under 40 years old (*P* = 0.01, OR: 1.57(1.10, 2.23)) (Table 3).

**Table 3:** Multiple logistic Regression model of factors related to RACP

Variable	Categories	OR	95% CI for OR	Sig
Education	Diploma	Reference category		
	Bachelor's	.984	(0.654, 1.479)	.93
	Master's Degree/ Ph.D	3.178	(1.672, 6.043)	.00
Age	40 >	Reference category		
	> 40	1.571	(1.103, 2.238)	.01

## Discussion

This study aimed to investigate the factors associated with RACP in Iranian society. In the present study the mean score of RACP was 104.46 ± (28.37). The results of the multiple logistic Regression Model indicated a significant relationship between age and RACP (*p* = 0.01), as such people over the age of 40 had higher RACP. On the other hand, the results showed that the chance of RACP in those over the age of 40 was 1.5-fold higher than those under 40 years. Additionally, there was a significant relationship between education level and RACP (*p* = 0.00).

According to the results of the present study, there was a significant relationship between age and RACP (*p* = 0.00), so that people over 40 years of age had higher RACP. On the other hand, the results showed that the high readiness of those over 40 years old for ACP was 1.5-fold higher than those less than 40 years old. In line with the current study, the results of the study by Wang et al. [34] that showed a moderate RACP in participants, as well as Waller et al. [43] and Hong et al. [44] indicated that older people have a greater levels of RACP. This is due to the fact that while people are approaching their final years of life, they are more likely to plan for their end-of-life care. Older people due to their age-associated characteristics have higher sense of completion of life compared with younger people and therefore seem to be more comfortable to accept ACP for end of life.

In the present study, there was a significant relationship between the level of education and RACP (*P* = 0.00). Consistent with the results of the present study, the study conducted by Black et al. in the USA, also revealed that the level of readiness for end-of-life care increases by education level [26]. In addition, in a study performed by Spelten et al. in 2019, education level and social support were regarded as influencing factors on RACP, as such RACP increases in those with higher levels of education and social support [45].

In the studies carried out by Hong et al. (2021) and Yap et al. [28] the role of education and knowledge was also noted as an effective factor in increasing RACP. The higher levels of education and awareness shown to have a positive correlation with individuals’ RACP [28, 44]. In contrast with our study, the study by Fraser et al. demonstrated that merely high education cannot be effective in preparing people for end-of-life care. This contrast can be due to the difference in the research population (final year of medical students) [46]. Evidence suggests that people with higher levels of education often have a deeper understanding about the importance and the process of ACP [44, 47]. This in part is due to greater access to the information and the ability to benefit from educational resources concerning end-of-life care. This makes them more motivated and prepared to participate in the preventive planning process, the end-of-life care or ACP.

In this study, no significant difference was found between RACP with gender, marital status, income, living condition, employment status and loss of loved ones or family caregiving. These results were consistent with the results of the study conducted by Zhang et al., which demonstrated low levels of awareness about RACP [41]. In addition, Black et al. [26] and Wachterman et al. [48], found that women have a higher level of RACP. Tang et al. [49] also found that married people are more likely to discuss the end–of–life care process with their doctor compared to single people [49]. The studies by Fitzpatrick et al. [50] and Carr et al. [51], also showed that the participants’ job category and economic advantage could be effective in planning for end-of-life care indicating that people with high-paying jobs are more likely to consider end-of-life care [52, 53]. Besides, it has been pointed out that the likelihood of readiness for end-of-life care reduces in those who live alone [27]. Another study found that people who experienced loss or family caregiving are more likely to plan for end-of-life care [54]. The reasons for this disparity can be the difference between the research population, culture, rules and regulations that govern the society, study method, and the assessed variables.

## Conclusion

According to the findings of this study, it can be concluded that there is relative RACP among Iranian society. Discovering characteristics associated with RACP can enable health care providers to tailor discussions with people. In this study, age and level of education are factors influencing people for RACP, so that people's RACP increases with their age and level of education. Therefore, considering the relative readiness of the Iranian society to receive advanced care planning, it seems that the time has come for serious measures to be taken at the levels of legislation, policy making, education and implementation of advanced care planning in Iran. Therefore, public RACP can be increased to improve end-of-life outcome by holding appropriate programmatic and information sessions.

## Limitation

Considering that the concept of ACP is very new in Iran, one of the limitations of this study was the unfamiliarity of the population with this concept and their poor cooperation and participation in this study. The researchers have had to provide long, detailed explanations of the nature and goals of ACP to the participants in order to attract their participation and cooperation. Also, this study was a cross-sectional study, hence, we were not able to determine causal relationship between variables. In addition, the research population was restricted to a specific geographic region. Therefore, to improve the generalizability of the study results to the entire Iranian society, considering larger sample size and using random sampling methods is required.

### Suggestions for further studies

Given the importance of advanced preparedness of individuals for their future medical care, following acts are recommended: self-organizing workshops and public training sessions about ACP in order to increase public awareness and knowledge, designing and implementing training programs tailored to the needs of different gender and age groups, empowering physicians and nurses to start discussing ACP with patients, as well as providing free legal and financial advice to assist individuals with the completion of legal documents related to ACP. Finally, further studies seem to be necessary to identify cultural barriers associated with ACP in Iranian society and find solutions to deal with them.

## Supplementary Information

Supplementary Material 1.

## Data Availability

No datasets were generated or analysed during the current study.
