# Correction to: Readiness for advance care planning and related factors in the general population: a cross sectional study in Iran

**DOI:** 10.1186/s12904-024-01553-w

**Published:** 2024-09-30

**Authors:** Ali Askari, Hosein Mohammadi Roshan, Nasim Abbaszadeh, Mahmood Salesi, Seyed Morteza Hosseini, Mobina Golmohammadi, Salman Barasteh, Omid Nademi, Razieh Mashayekh, Mohammad Hossein Sadeghi

**Affiliations:** 1 Medicine, Quran and Hadith Research Center, Baqiyatallah University of Medical Sciences, Tehran, Iran; 2 Nursing Faculty, Baqiyatallah University of Medical Sciences, Tehran, Iran; 3 Student Research Committee, Baqiyatallah University of Medical Sciences, Tehran, Iran; 4 Chemical Injuries Research Center, Systems Biology and Poisonings Institute, Baqiyatallah University of Medical Sciences, Tehran, Iran; 5 Nursing Care Research Center, Clinical Sciences Institute, Baqiyatallah University of Medical Sciences, Tehran, Iran

**Correction to: BMC Palliat Care 23, 167 (2024).**

10.1186/s12904-024-01496-2

Following publication of the original article [1], the author reported that the data in Table 2 in line with „Death Experience“ were not correctly placed in the PDF but correct in the XML.

The incorrect presentation is:

**Table 2:**
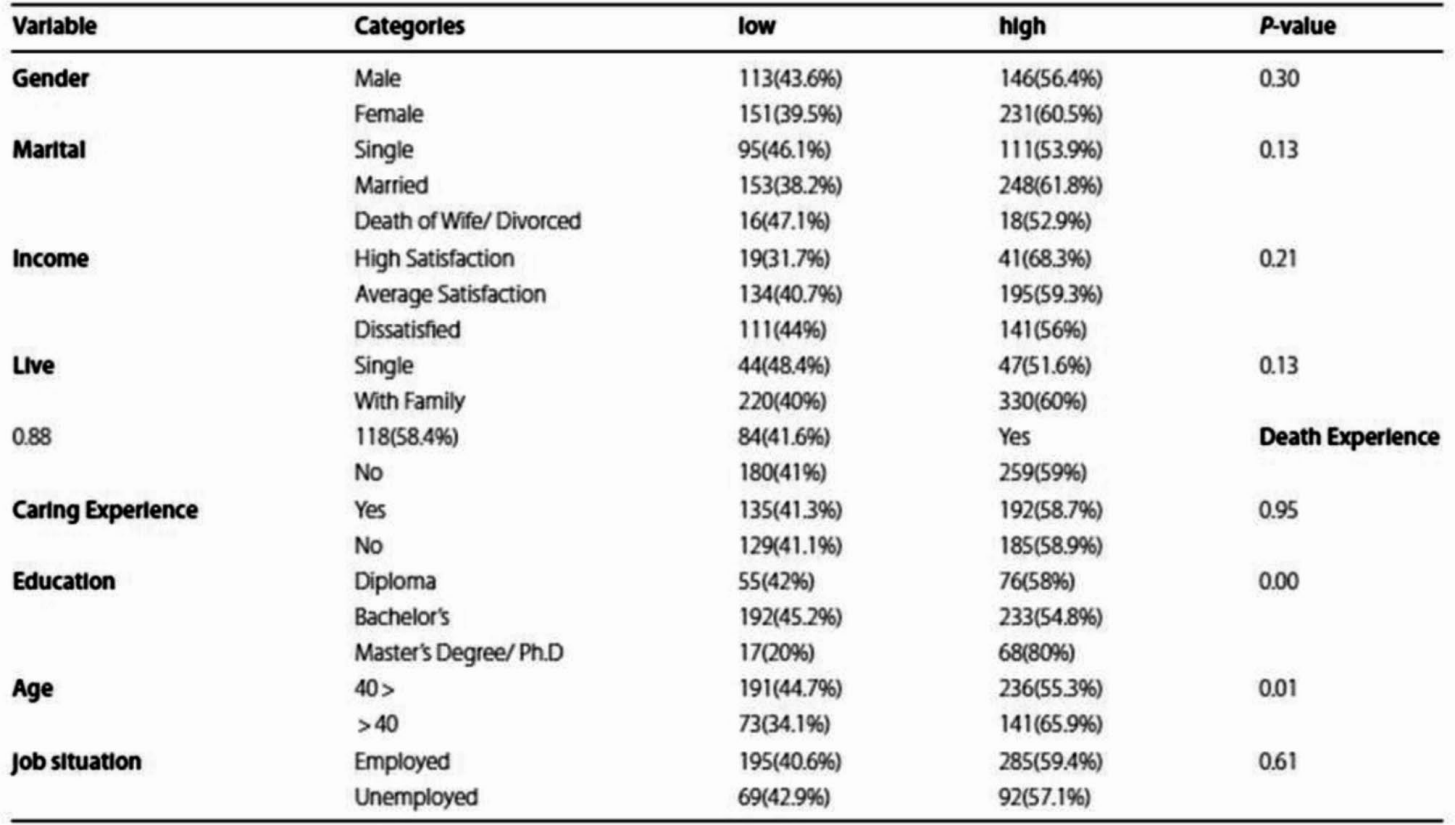
Correlations between variables and RACP

The correct presentation should be:

**Table 2:** Correlations between variables and RACP

Variable	Categories	Low	High	*P*-value
**Gender**	Male	113(43.6%)	146(56.4%)	0.30
	Female	151(39.5%)	231(60.5%)	
**Marital**	Single	95(46.1%)	111(53.9%)	0.13
	Married	153(38.2%)	248(61.8%)	
	Death of Wife/ Divorced	16(47.1%)	18(52.9%)	
**Income**	High Satisfaction	19(31.7%)	41(68.3%)	0.21
	Average Satisfaction	134(40.7%)	195(59.3%)	
	Dissatisfied	111(44%)	141(56%)	
**Live**	Single	44(48.4%)	47(51.6%)	0.13
	With Family	220(40%)	330(60%)	
0.88	118(58.4%)	84(41.6%)	Yes	**Death Experience**
	No	180(41%)	259(59%)	
**Caring Experience**	Yes	135(41.3%)	192(58.7%)	0.95
	No	129(41.1%)	185(58.9%)	
**Education**	Diploma	55(42%)	76(58%)	0.00
	Bachelor’s	192(45.2%)	233(54.8%)	
	Master’s Degree/Ph.D	17(20%)	68(80%)	
**Age**	40 >	191(44.7%)	236(55.3%)	0.01
	> 40	73(34.1%)	141(65.9%)	
**job situation**	Employed	195(40.6%)	285(59.4%)	0.61
	Unemployed	69(42.9%)	92(57.1%)	

The original article has been updated to correct the PDF.

## References

[1] Askari A, Roshan HM, Abbaszadeh N, et al. Readiness for advance care planning and related factors in the general population: a cross sectional study in Iran. BMC Palliat Care. 2024;23:167. 10.1186/s12904-024-01496-2. 38982407 10.1186/s12904-024-01496-2 PMC11234553

