# Supplementary material for: Readiness for advance care planning and related factors in the general population: a cross sectional study in Iran
Source: BMC Palliat Care. 2024 Jul 9;23:167. doi: 10.1186/s12904-024-01496-2 (PMC11234553; doi:10.1186/s12904-024-01496-2)
Supplement: Supplementary file 1 — Supplementary Material 1. [file 12904_2024_1496_MOESM1_ESM.docx]

| Response6(%) | Response5(%) | Response4(%) | Response3 | Response2 | response1 |  |
| --- | --- | --- | --- | --- | --- | --- |
| 121(18.9) | 149(23.2) | 126(19.7) | 76 | 72 | 97 | Q1 |
| 105(16.4) | 157(24.5) | 126(19.7) | 115 | 58 | 80 | Q2 |
| 1121(17.5) | 140(21.8) | 164(25.6) | 99 | 78 | 48 | Q3 |
| 98(23.7) | 159(24.8) | 170(26.5) | 100 | 63 | 51 | Q4 |
| 152(23.7) | 159(24.8) | 127(19.8) | 99 | 68 | 36 | Q5 |
| 158(24.6) | 161(25.1) | 149(23.2) | 80 | 50 | 43 | Q6 |
| 169(26.4) | 181(28.2) | 136(21.2) | 63 | 51 | 41 | Q7 |
| 122(19) | 106(16.5) | 130(20.3) | 106 | 96 | 81 | Q9 |
| 119(18.6) | 148(23.1) | 149(23.2) | 108 | 65 | 52 | Q10 |
| 126(19.7) | 147(22.9) | 146(22.8) | 106 | 61 | 55 | Q11 |
| 122(19) | 156(24.3) | 134(20.9) | 99 | 82 | 48 | Q12 |
| 112(17.5) | 112(17.5) | 157(24.5) | 110 | 76 | 74 | Q13 |
| 100(15.6) | 117(18.3) | 159(24.8) | 105 | 85 | 75 | Q14 |
| 124(19.3) | 127(19.8) | 130(20.3) | 105 | 84 | 71 | Q15 |
| 122(19) | 112(17.5) | 149(23.2) | 98 | 83 | 77 | Q16 |
| 132(20.6) | 145(22.6) | 145(22.6) | 83 | 78 | 58 | Q17 |
| 123(19.2) | 153(23.9) | 161(25.1) | 100 | 61 | 43 | Q18 |
| 107(16.7) | 138(21.5) | 147(22.9) | 104 | 81 | 64 | Q19 |
| 85(13.3) | 137(21.4) | 161(25.1) | 103 | 88 | 67 | Q20 |
| 52(8.1) | 84(13.1) | 132(20.6) | 118 | 124 | 131 | Q21 |
| 45(7) | 95(14.8) | 123(19.2) | 128 | 121 | 129 | Q22 |
| 55(8.6) | 90(14) | 124(19.3) | 113 | 109 | 150 | Q23 |
| 83(12.9) | 115(17.9) | 122(19) | 116 | 108 | 97 | Q24 |
| 68(10.6) | 95(14.8) | 106(16.5) | 105 | 127 | 140 | Q25 |
| 59(9.2) | 95(14.8) | 101(15.8) | 99 | 126 | 161 | Q26 |
| 63(9.8) | 91(14.2) | 106(16.5) | 98 | 121 | 162 | Q27 |
| 113(17.6) | 125(19.5) | 118(18.4) | 95 | 86 | 104 | Q28 |
